# Supplementary material for: Glibenclamide-Loaded Engineered Nanovectors (GNVs) Modulate Autophagy and NLRP3-Inflammasome Activation
Source: Pharmaceuticals (Basel). 2023 Dec 13;16(12):1725. doi: 10.3390/ph16121725 (PMC10747596; doi:10.3390/ph16121725)
Supplement: Supplementary file 1 [file pharmaceuticals-16-01725-s001.zip › pharmaceuticals-2739617-supplementary.pdf]

Table 1: Primers

**NLRP3**	**F**	**AGGTGTTGGAATTAGACAAC**
	**R**	**AATACATTTCAGACAACCCC**
**beclin-1**	**F**	**ATCTCGAGAAGGTCCAGGCT**
	R	CTGTCCACTGTGCCAGATGT
**LC3**	F	CAGCATCCAACCAAAATCCC
	R	GTTGACATGGTCAGGTACAAG
**p62**	F	CCAGAGAGTTCCAGCACAGA
	R	CCGACTCCATCTGTTCCTCA
**lamp2A**	F	GCAGTGCAGATGAAGACAAC
	R	AGTATGATGGCGCTTGAGAC
**hsc70**	F	CAGGTTTATGAAGGCGAGCGTGCC
	R	GGGTGCAGGAGGTATGCCTGTGA
**β-actin**	F	TGTGGCATCCACGAAACTAC
	R	GGAGCAATGATCTTGATCTTCA
